# Exploiting bacterial outer membrane vesicles as a cross-protective vaccine candidate against avian pathogenic *Escherichia coli* (APEC)

**DOI:** 10.1186/s12934-020-01372-7

**Published:** 2020-06-03

**Authors:** Rujiu Hu, Jing Li, Yuezhen Zhao, Hua Lin, Liu Liang, Mimi Wang, Haojing Liu, Yuna Min, Yupeng Gao, Mingming Yang

**Affiliations:** 1 College of Animal Science and Technology, Northwest A&F University, Yangling, 712100 Shaanxi China; 2 Department of Animal Engineering, Yangling Vocation and Technical College, Yangling, 712100 Shaanxi China

**Keywords:** Avian pathogenic *E. coli* (APEC), Outer membrane vesicles, Vaccine, Cross-protection, Immune response, Multi-serogroup

## Abstract

**Background:**

The well-known fact that avian pathogenic *Escherichia coli* (APEC) is harder to prevent due to its numerous serogroups has promoted the development of biological immunostimulatory materials as new vaccine candidates in poultry farms. Bacterial outer membrane vesicles (OMVs), known as spherical nanovesicles enriched with various immunostimulants, are naturally secreted by Gram-negative bacteria, and have gained much attention for developing effective vaccine candidates. Recent report has demonstrated that OMVs of APEC O78 can induce protective immunity in chickens. Here, a novel multi-serogroup OMVs (MOMVs) vaccine was developed to achieve cross-protection against APEC infection in broiler chickens.

**Results:**

In this study, OMVs produced by three APEC strains were isolated, purified and prepared into MOMVs by mixing these three OMVs. By using SDS-PAGE and LC–MS/MS, 159 proteins were identified in MOMVs and the subcellular location and biological functions of 20 most abundant proteins were analyzed. The immunogenicity of MOMVs was evaluated, and the results showed that MOMVs could elicit innate immune responses, including internalization by chicken macrophage and production of immunomodulatory cytokines. Vaccination with MOMVs induced specific broad-spectrum antibodies as well as Th1 and Th17 immune responses. The animal experiment has confirmed that immunization with an appropriate dose of MOMVs could not cause any adverse effect and was able to reduce bacteria loads and pro-inflammatory cytokines production, thus providing effective cross-protection against lethal infections induced by multi-serogroup APEC strains in chickens. Further experiments indicated that, although vesicular proteins were able to induce stronger protective efficiency than lipopolysaccharide, both vesicular proteins and lipopolysaccharide are crucial in MOMVs-mediated protection.

**Conclusions:**

The multi-serogroup nanovesicles produced by APEC strains will open up a new way for the development of next generation vaccines with low toxicity and broad protection in the treatment and control of APEC infection.

## Background

*Escherichia coli* (*E. coli*) is a commensal bacteria in human and animal intestine as well as a common zoonotic pathogen. Avian pathogenic *E. coli* (APEC) refers to *E. coli* strains that can cause extraintestinal diseases in chicken and other avian species [1]. As a major bacterial pathogen in the poultry industry worldwide, APEC can cause typical colibacillosis in broiler chickens, such as colisepticemia, granuloma, air sacculitis, pericarditis and cellulitis [2]. APEC can infect chickens of different types and ages and lead to high morbidity and mortality rates in young chickens, resulting in huge economic losses every year [3]. Furthermore, a number of studies have shown that APEC may act as a human pathogen because they share some homologous virulence genes with human extraintestinal pathogenic *E. coli* [4–6]. Currently, prevention and treatment strategies of avian colibacillosis are commonly conducted based on the use of antibiotics. However, with the gradual prohibition of antimicrobial drugs in animal husbandry and the emergence of multidrug-resistant bacteria, it becomes difficult and costly to control APEC infection [7]. In addition, drug residues and resistant gene transfer may pose a great threat to human health [8]. Hence, it is urgently needed to search alternative preventive strategies to ameliorate APEC infection.

Vaccination is considered the most effective and economical means of controlling infectious diseases. Many vaccine candidates have been developed against APEC infection in chickens, including inactivated, live attenuated and subunits vaccines [9]. Inactivated vaccines were initially developed by killing the live whole-bacteria, which have not been widely used because of their low protective efficacy. Live attenuated APEC vaccines can provide stronger protection than inactivated vaccines. However, they have many obvious disadvantages, such as poor safety and short-term protection. As for the subunit vaccines, although they are generally safe, their application limited due to the high cost and complicated production process [10]. Moreover, these vaccines are not able to provide effective cross-protection against infections induced by multi-serogroup APCE strains [1, 9]. Since APCE strains have numerous serogroups and are widely distributed, an effective cross-protective vaccine is needed for broad-spectrum protection.

Vaccines based on outer membrane vesicles (OMVs) have gained increasing attention for preventing bacterial infections. OMVs are spherical vesicles with a bilayered proteolipid structure, which are naturally secreted by Gram-negative bacteria [11]. These vesicles contain immunoactive molecules, including cell-wall components, membrane proteins, cytoplasmic proteins and bacterial nucleic acids [12]. Some of these components nanosized are capable of eliciting antigen-specific immune responses [13, 14]. Because of the highly biocompatible nanosized structures and the naturally enriched immunogenic components, bacterial OMVs are widely considered as promising candidates for the next generation vaccine. Recent studies have shown that OMVs derived from many Gram-negative bacteria, such as *Pseudomonas aeruginosa* [15], *Salmonella typhimurium* [16], *Klebsiella pneumoniae* [17], and *Shigellae* [18], are able to induce strong protective immunity in animal models of bacterial infection. Furthermore, several studies have suggested that vaccination with OMVs confers cross-protection against many serogroups of the same pathogen [15, 19]. *Neisseria meningitides*-derived OMVs vaccine has been licensed worldwide for controlling meningococcal B disease in humans [20].

OMVs produced by *E. coli* (OMV_EC_) have been observed in many studies [21–23]. Various heterogeneous cargoes, including virulence factors, immunomodulatory factors and quorum-sensing signaling molecules, were identified in OMV_EC_, indicating that the vesicles are associated with the physiology and pathogenesis of the bacteria [24, 25]. The protective immunity of OMV_EC_ also has been confirmed in mouse model of bacterial infection [22, 26]. Hence, we reasonably speculated that OMVs produced by APEC (OMV_APEC_) could be used as the candidate antigens for vaccines against APEC infection. Recently, Wang and colleagues demonstrated that the single serogroup OMVs of APEC O78 can induce protective immunity against APEC O78 infection in chickens [27]. However, due to the diversity of APEC serogroups, it may be difficult to achieve 100% protective efficacy against multi-serogroups with a single serogroup OMV_APEC_. Among the known serogroups of APEC, O1, O2 and O78 are predominantly associated with chicken colibacillosis outbreaks across the world [1, 9]. Thus, the majority of APEC infection could be controlled by a cross-protective vaccine against these three serogroups.

Therefore, in the current study, we tried to obtain purified OMV_APEC_ from three APEC serogroups, and then develop a novel multi-serogroup OMV_APEC_ (MOMVs) vaccine by formulating a mixed immunogen with these three different OMV_APEC_. We investigated the safety and immunogenicity of the MOMVs as well as the cross-protective effect of MOMVs vaccination in the chicken model of APEC infection. Our goal is to use OMVs produced by natural APEC strains for the development of practical broad-spectrum vaccines against multi-serogroup APEC infections.

## Results

### Preparation and characterization of MOMV*s*

**Fig. 1:**
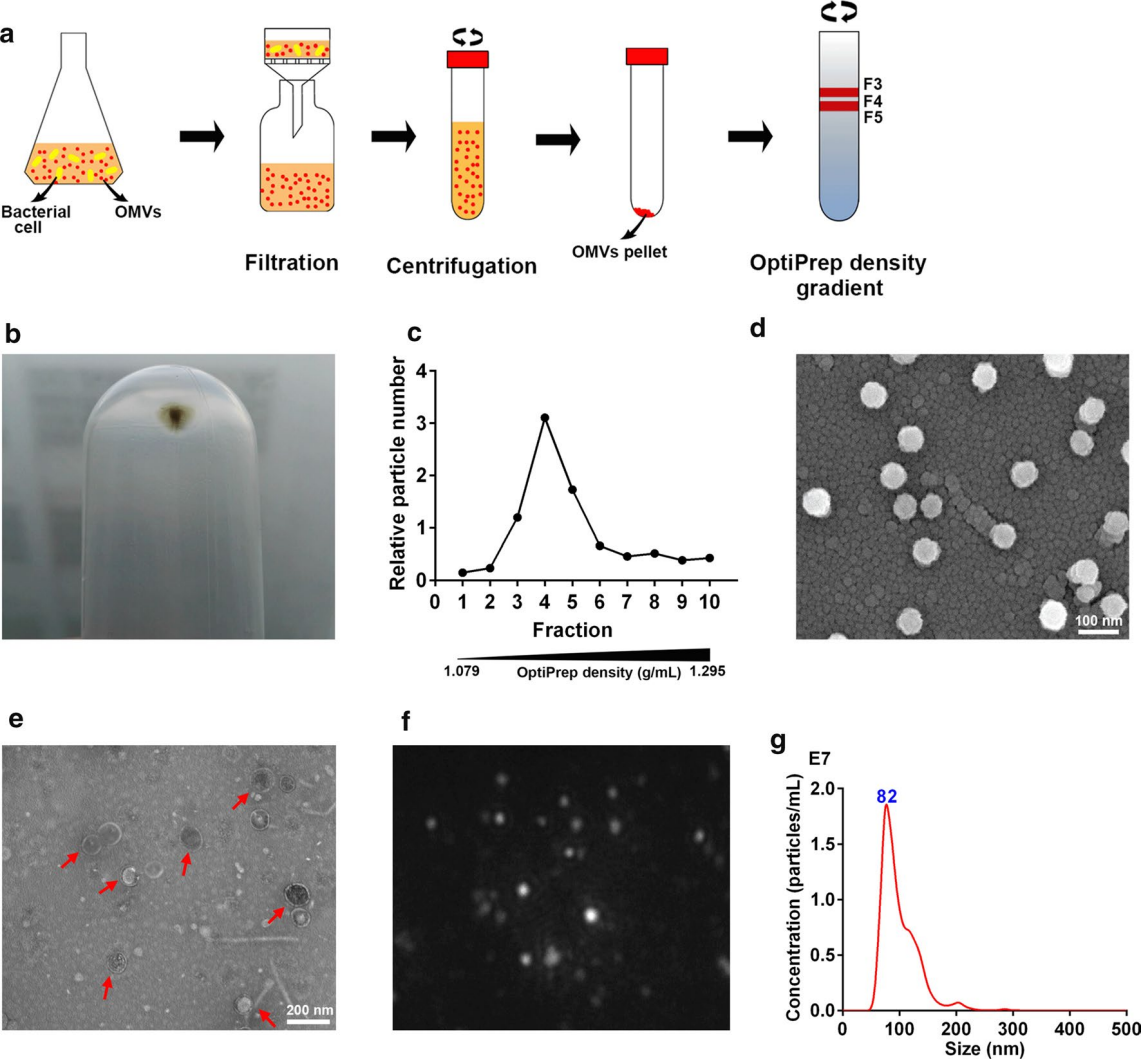
Preparation and visualization of OMVs derived from avian pathogenic *Escherichia coli*. **a** Isolation and purification protocols of bacterial OMVs. **b** Native OMVs were isolated and pelleted by ultracentrifugation. **c** OMVs were purified by Optiprep density gradient ultracentrifugation. The particle numbers of the resulting fractions (1–10) were detected by nanoparticle tracking analysis (NTA). Purified MOMV_S_ were visualized using scanning electron microscope (**d**) and transmission electron microscopy (**e**) after negative staining. **f** Representative frame was captured from the MOMVs NanoSight videos. **g** Size distribution and concentration of these vesicles was determined by NTA

Isolation and purification procedures of bacterial OMVs are shown in Fig. 1a. A large vesicle pellet (Fig. 1b) was obtained from the culture supernatant of APEC strain using ultracentrifugation. After purification by density gradient centrifugation, the vast majority of particles were detected in fractions 3–5 using NTA (Fig. 1c). The densities of these fractions range from 1.127 to 1.175 g/mL, which is consistent with the previously reported density of bacterial OMVs [26]. Purified MOMVs were made with each purified OMVs from three APEC strains and observed by scanning electron microscopy (Fig. 1d) and transmission electron microscopy (Fig. 1e). The results show that the APEC strains abundantly produced the spherical vesicles with a morphology of bilayer membrane. Typical results from NTA characterization of MOMVs, as shown in Fig. 1f and g, reveal that the sizes of the majority of MOMVs range from 50 to 200 nm and peak at 82 nm, which is in accordance with the previously determined sizes of bacterial OMVs [23].

### Proteomic analysis of MOMVs

**Fig. 2:**
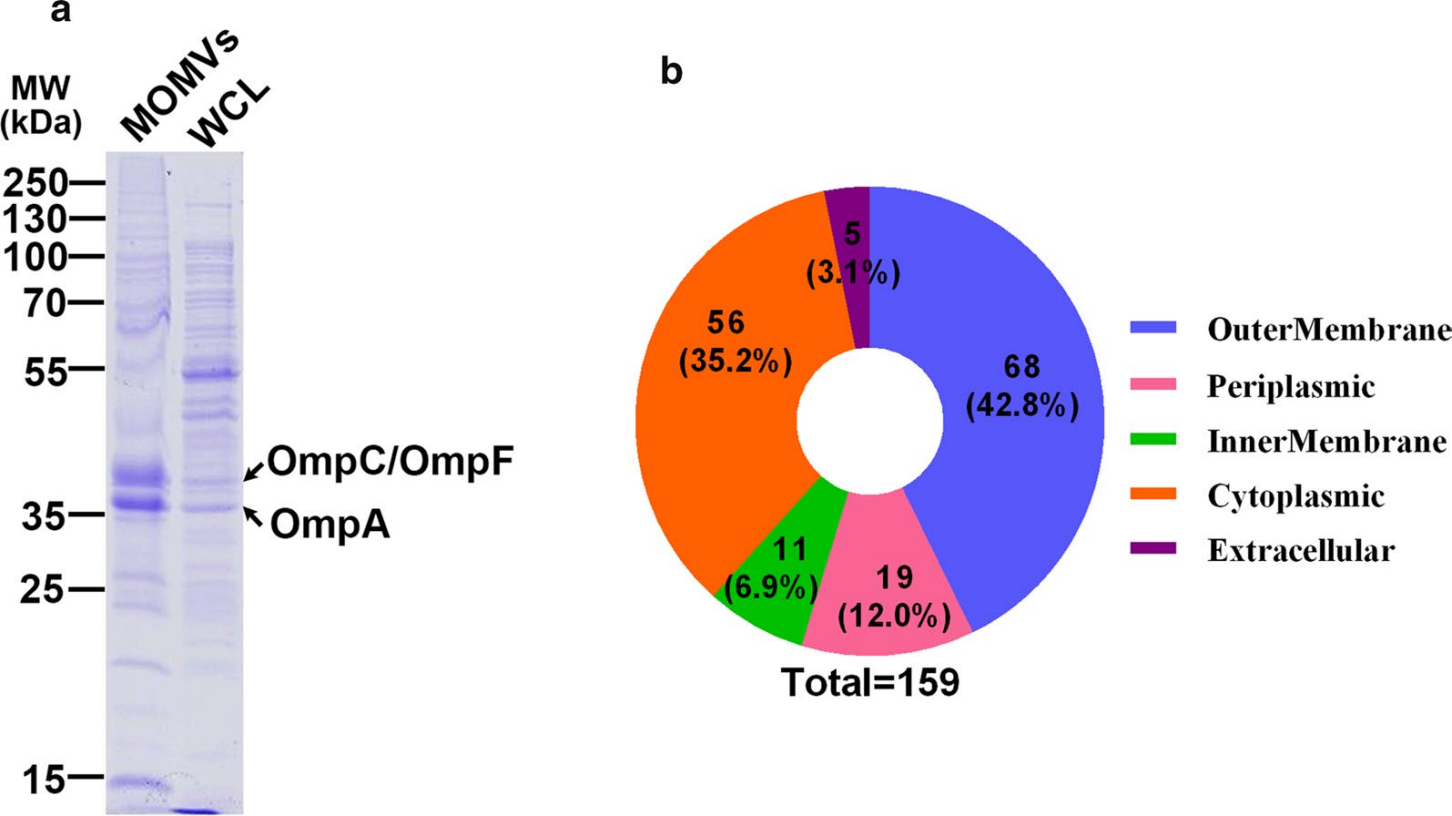
Proteomic analysis of MOMVs derived from APEC strains. **a** Protein profiles of MOMVs and whole-cell lysates (WCL) from three APEC strains analyzed by Coomassie Brilliant Blue-stained SDS-PAGE. Arrows represent the two major vesicular protein bands. **b** Proteins of MOMVs identified by LC–MS/MS were classified according to their subcellular localizations

SDS-PAGE analyses of proteins from MOMVs and whole cell lysates are shown in Fig. 2a. Several OMPs (e.g., OmpA, OmpC and OmpF) and lipoproteins were found in the lane of the MOMVs samples according to the molecular weight. The protein composition was confirmed by the subsequent LC–MS/MS analysis. By proteomic analysis, 159 proteins were identified, and their subcellular localization are shown in Fig. 2b. Of these identified proteins, 68 (42.8%), 56 (35.2%), 19 (12.0%) and 11 (6.9%) were derived from outer membrane, cytoplasm, periplasm and inner membrane, respectively. The 20 most abundant proteins found in MOMVs, their subcellular localization and biological functions are shown in Table 1. Many OMPs, such as OmpA, OmpC, OmpX and OmpW, and some outer membrane lipoproteins were highly enriched, suggesting that MOMVs and outer membrane of their parental bacteria have high similarity in function and structure. Furthermore, the abundance of 60 kDa chaperonin, Fe (3+) dicitrate transport protein, LPS-assembly protein and ferrichrome outer membrane transporter indicated functions of the MOMVs involved in protein processing, virulence, signaling, etc. These multiple immunogenic proteins found in MOMVs suggested that MOMVs had the potential to be an effective vaccine candidate.

**Table 1:** Top 20 most abundant proteins identified in MOMVs

Rank	Protein accession	Gene name	Protein annotation	Subcellular localization	Biological function	MW (kDa)	Intensity
1	P0A910	ompA	Outer membrane protein A	Outer membrane	Cell wall/membrane/envelope biogenesis	37.2	7.57E+11
2	P69776	lpp	Major outer membrane prolipoprotein Lpp	Outer membrane	Cell wall/membrane/envelope biogenesis	8.3234	6.39E+11
3	P06996	ompC	Outer membrane protein C	Outer membrane	Cell wall/membrane/envelope biogenesis	40.368	4.83E+11
4	P0A903	bamC	Outer membrane protein assembly factor BamC	Outer membrane	Cell wall/membrane/envelope biogenesis	36.842	3.39E+11
5	P0A6F5	groL	60 kDa chaperonin	Cytoplasmic	Posttranslational modification, protein turnover, chaperones	57.328	3.07E+11
6	P0A905	slyB	Outer membrane lipoprotein SlyB	Outer membrane	Cell wall/membrane/envelope biogenesis	15.601	1.87E+11
7	P0A917	ompX	Outer membrane protein X	Outer membrane	Cell wall/membrane/envelope biogenesis	18.602	9.60E+10
8	P0A908	mipA	MltA-interacting protein	Outer membrane	Cell wall/membrane/envelope biogenesis	27.831	8.14E+10
9	P61320	lolB	Outer-membrane lipoprotein LolB	Outer membrane	Cell wall/membrane/envelope biogenesis	23.55	6.45E+10
10	P0A915	ompW	Outer membrane protein W	Outer membrane	Cell wall/membrane/envelope biogenesis	22.928	5.63E+09
11	P06959	aceF	Dihydrolipoyllysine-residue acetyltransferase component of pyruvate dehydrogenase complex	Cytoplasmic	Energy production and conversion	66.095	5.45E+09
12	P09394	glpQ	Glycerophosphodiester phosphodiesterase	Periplasmic	Energy production and conversion	40.843	4.41E+09
13	P13036	fecA	Fe (3 +) dicitrate transport protein FecA	Outer membrane	Inorganic ion transport and metabolism	85.321	4.02E+09
14	P0A940	bamA	Outer membrane protein assembly factor BamA	Outer membrane	Cell wall/membrane/envelope biogenesis	90.552	2.09E+09
15	P31554	lptD	LPS-assembly protein LptD	Outer membrane	Cell wall/membrane/envelope biogenesis	89.67	9.78E+08
16	P06971	fhuA	Ferrichrome outer membrane transporter/phage receptor	Outer membrane	Inorganic ion transport and metabolism	82.181	7.20E+08
17	P21420	nmpC	Putative outer membrane porin protein NmpC	Outer membrane	Cell wall/membrane/envelope biogenesis	40.302	7.03E+08
18	P0AFG8	aceE	Pyruvate dehydrogenase E1 component	Cytoplasmic	Energy production and conversion	99.667	6.63E+08
19	P21513	rne	Ribonuclease E	Cytoplasmic	Translation, ribosomal structure and biogenesis	118.2	6.48E+08
20	P00968	carB	Carbamoyl-phosphate synthase subunit beta	Periplasmic	Membrane biogenesis/synthesize carbamoyl phosphate	117.842	4.28E+08

MOMVs represents multi-serogroup outer membrane vesicles derived from avian pathogenic *Escherichia coli*

### MOMVs induced innate immune responses in vitro

**Fig. 3:**
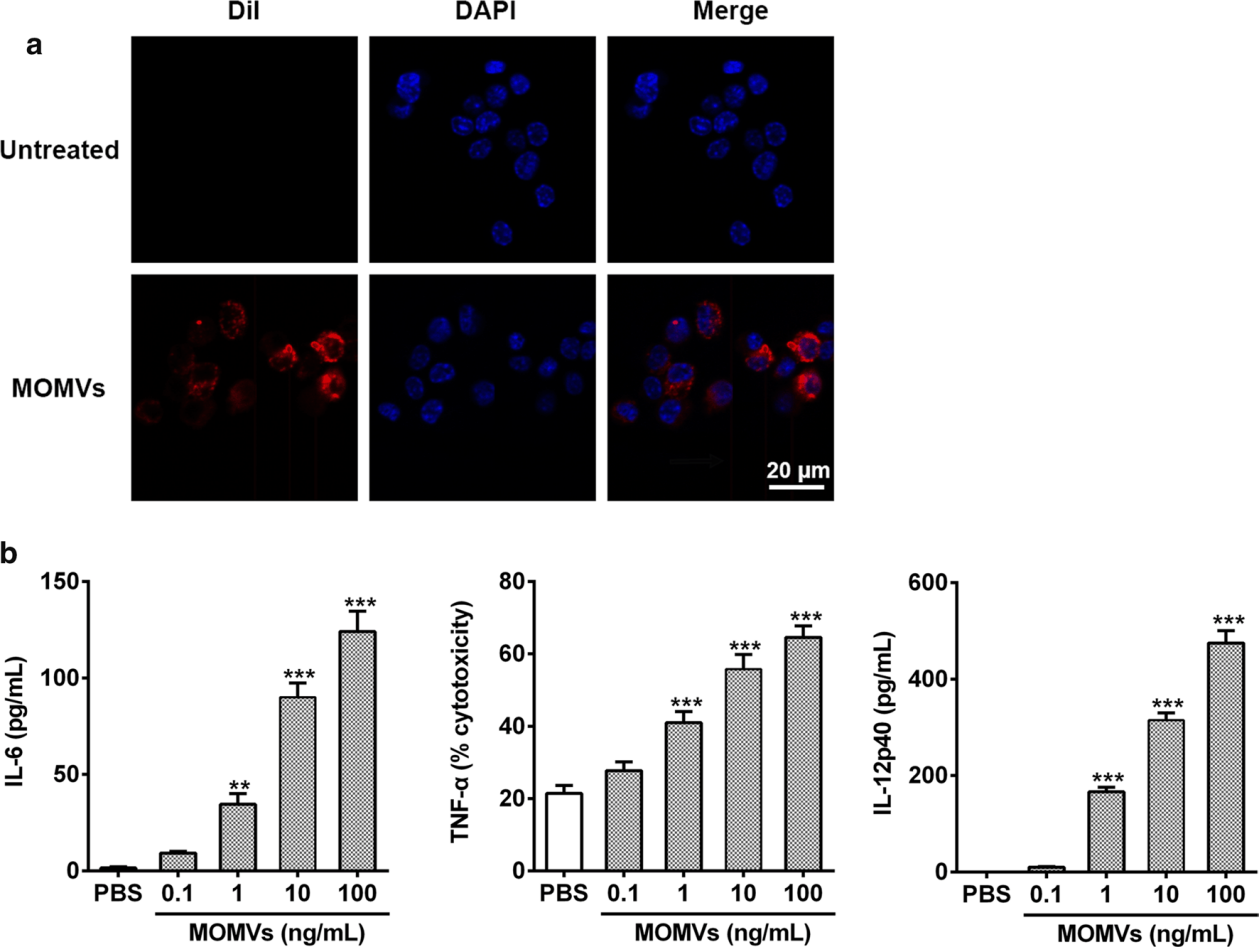
MOMVs were internalized by chicken HD11 cells and induced innate immune responses. **a** HD11 cells were treated with medium (row 1) or MOMVs (row 2) for 6 h at 37 °C. MOMVs were stained with DiI (red), and the cell nucleus was stained with DAPI (blue). **b** The production of IL-6, TNF-α and IL-12p40 estimated from the supernatant of HD11 cells after stimulation with MOMVs for 16 (n = 5). IL-12p40 production was measured by ELISA kit, the production of IL-6 and TNF-α was estimated by IL-6 and TNF-α activity bioassays, respectively. IL-6 production is expressed as pg/mL supernatant, and TNF-α production was reported as percent specific cytotoxicity. Data are representative of three independent experiments. *P < 0.05; **P < 0.01; ***P < 0.001; versus the control (PBS)

To evaluate their potential immunogenicity, we first investigated whether chicken macrophages could recognize and respond to MOMVs in vitro. Confocal microscopy analysis showed that the red signals were found in the cytoplasm of chicken HD11 macrophages when these cells were treated with the DiI-labeled MOMVs, revealing that these vesicles were taken up by macrophages (Fig. 3a). Furthermore, HD11 cells stimulated with MOMVs secreted higher production of immunomodulatory cytokines in a dose-dependent manner, including IL-6 (Th17-polarizing cytokines), TNF-α (pro-inflammatory cytokines) and IL-12 (Th1-polarizing cytokines) (Fig. 3b). These findings indicated that MOMVs were effectively internalized by chicken macrophages and provoked the innate immune cells to produce cytokines that are able to mediate adaptive immune responses.

### Immunization with MOMVs provoked specific humoral immune responses

**Fig. 4:**
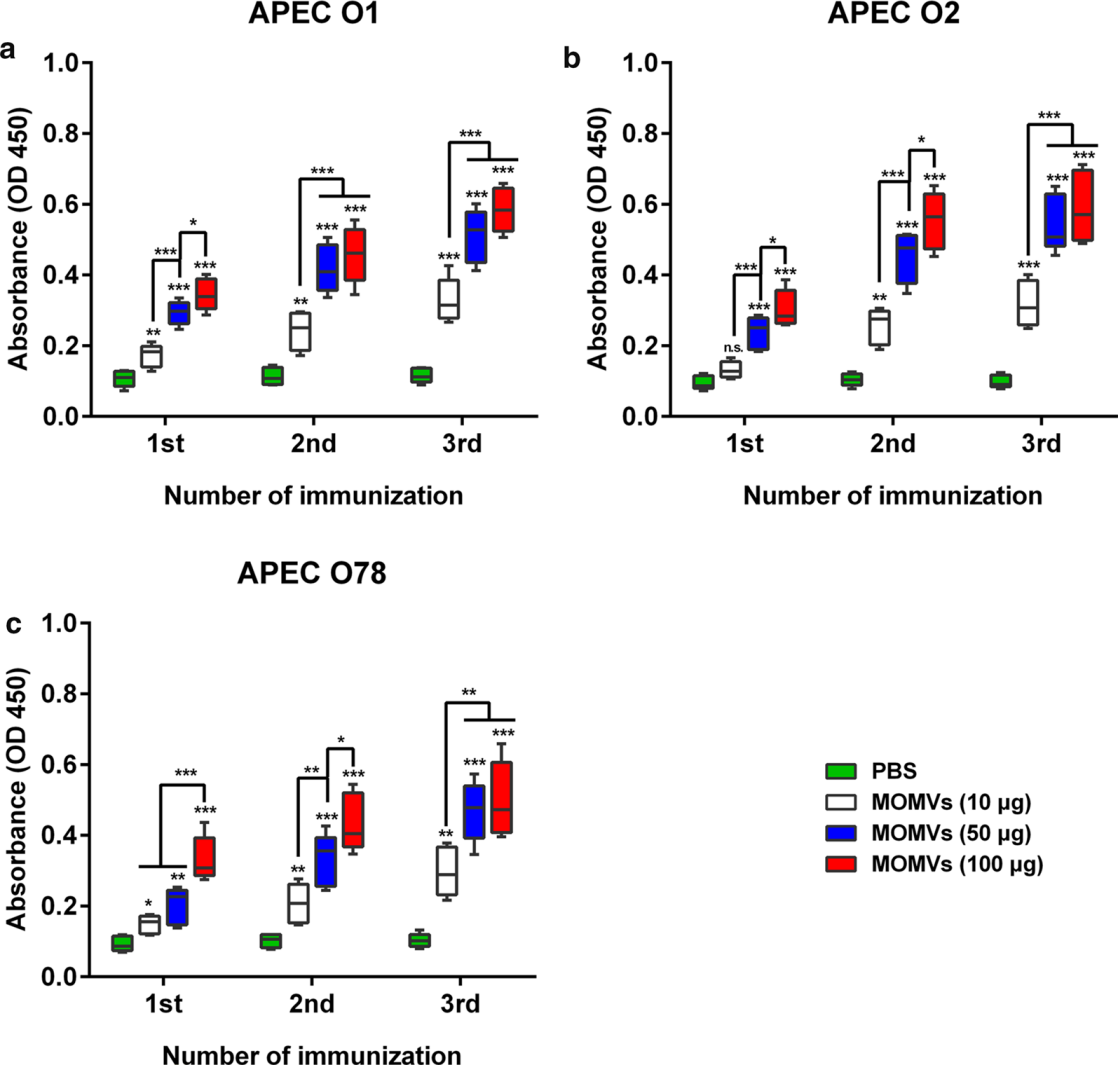
Immunization with MOMVs provoked specific antibody responses against each OMVs of these three OMVs. The production of specific IgG in MOMVs- and PBS-immunized sera was determined against each OMVs, respectively: **a** OMVs derived from APEC O1; **b** OMVs derived from APEC O2 ser; **c** OMVs derived from APEC O78. Sera were sampled from each group (n = 5) 7 days after the first, second and third immunization. The production of specific IgG was measured by ELISA. Data are representative of two independent experiments. *P < 0.05; **P < 0.01; ***P < 0.001; versus the control (PBS)

To further test the immunogenicity of MOMVs, we also evaluated the effect of MOMVs vaccination on the induction of specific IgG titers against these three OMVs in vivo. The production of specific IgG was determined for the first, second and third immunization. We found that immunization with MOMVs significantly improved the production of specific IgG against these three OMVs (Fig. 4a–c) in both dose-dependent and frequency-dependent manners. After the final immunization, the specific IgG production in the group immunized with 50 μg of MOMVs was not significantly different from that in the group immunized with 100 μg of MOMVs This result implied that immunization with 50 μg of MOMVs might be sufficient to induce a strong specific antibody response. Together, these results indicated that vaccination with MOMVs effectively induced specific antibody responses against each OMVs antigen.

### Immunization with MOMVs induced specific cellular immune responses

**Fig. 5:**
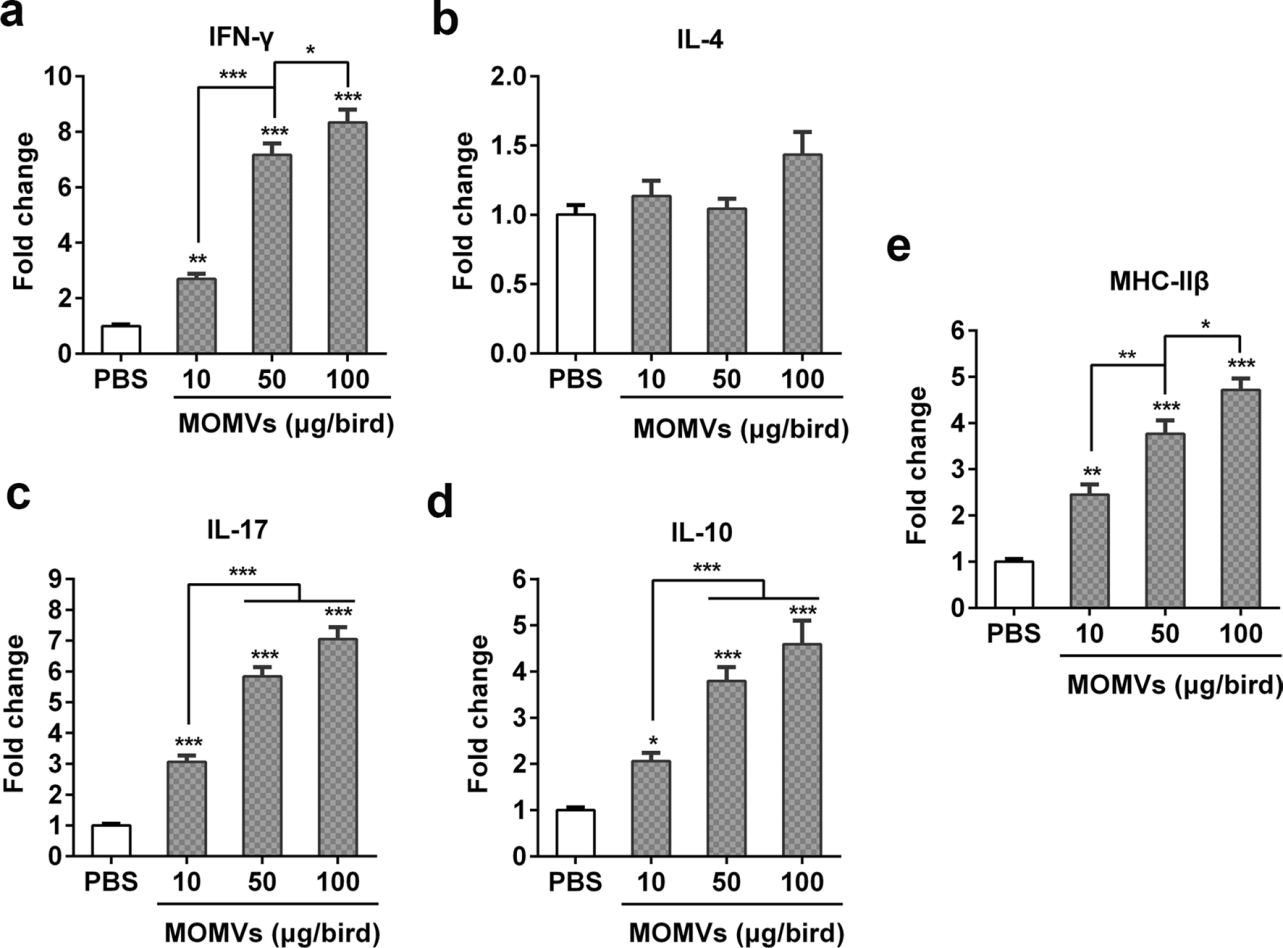
Immunization with MOMVs elicited cellular responses in spleen. One weeks after the final immunization, spleen tissues of chickens (n = 5) were sampled for the evaluation of immune gene expression. The qRT-PCR analysis was performed for the expression levels of cytokine genes: IFN-γ (**a**), IL-4 (**b**), IL-17 (**c**), IL-10 (**d**) and MHC-IIβ g (**e**). Data are representative of two independent experiments. *P < 0.05; **P < 0.01; ***P < 0.001; versus the control (PBS)

Next, we evaluated the effect of MOMVs immunization on the induction of cellular immune responses. One week after the final immunization, we determined the expression levels of major histocompatibility complex class II β gene (MHC-IIβ) and T cell-mediated immune genes, including T helper (Th)1 type cytokine (IFN-γ), Th2 type cytokine (IL-4), and regulatory T cell cytokine (IL-10) in spleen tissues. The results showed that IFN-γ (Fig. 5a) and IL-17 (Fig. 5c) were obviously activated in all MOMVs-immunized groups. However, the expression level of IL-4 gene (Fig. 5b) was similar in the MOMVs- and PBS-immunized groups. Moreover, the expression level of IL-10 (Fig. 5d), the major anti-inflammatory cytokine, was higher in the MOMVs-immunized groups compared with the control. These results indicated that both anti-inflammatory and pro-inflammatory cytokine genes were activated simultaneously in the MOMVs-immunized groups to balance the inflammatory responses. Furthermore, the higher expression level of MHC-IIβ (Fig. 5e), expressed in antigen-presenting cells (APCs), was also observed in the MOMVs-immunized groups. This finding suggested that MOMVs could be recognized by APCs, which in turn activate T cells. Collectively, these results suggested that immunization with MOMVs was able to activate APCs and elicit Th1- and Th17-cell responses.

### The immunization effect of MOMVs on cross-protection against APEC infections

**Fig. 6:**
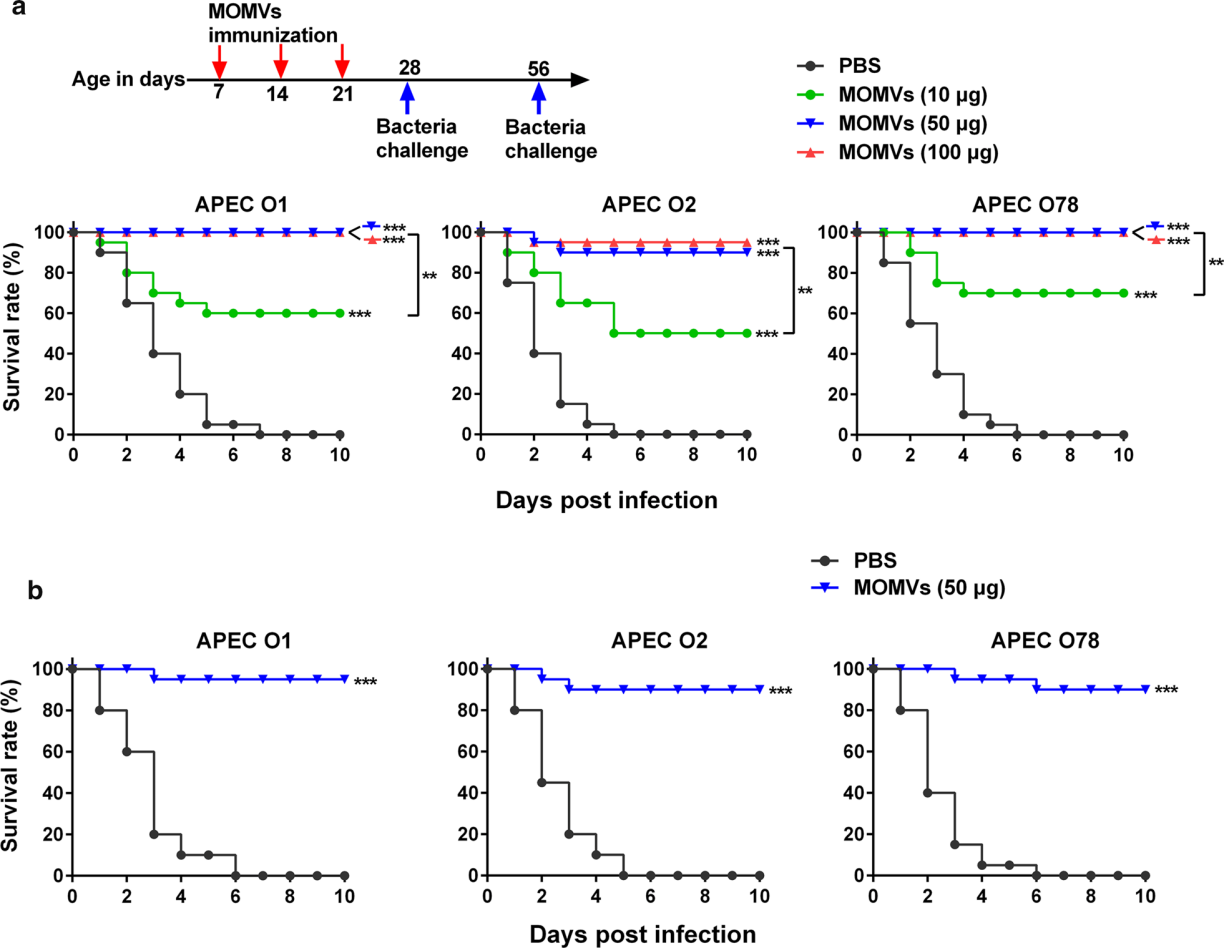
Immunization with MOMVs conferred cross-protection against infection of three APEC serogroups. **a** Survival rates of MOMVs- and PBS-immunized chickens after challenges of these three APEC serogroups (O1, O2 and O78). Chickens were immunized intramuscularly with MOMVs (10, 50 and 100 μg) or PBS at weekly intervals for 3 weeks (day 7, day 14 and day 21) (n = 20), and then challenged by the intratracheal route with the lethal dose (LD) of each APEC serogroup after the last immunization, respectively. **b** Survival rates of MOMVs (50 μg)- and PBS-immunized birds challenged with each APEC serogroup (2× LD) 5 weeks (day 56) after the last immunization (n = 10). The difference between each group was analyzed using the Kaplan–Meier method. Survival rate of each group was calculated every day for 10 days. **P < 0.01; ***P < 0.001; versus the control (PBS)

We first established chicken models of APEC infections by intratracheal injection of various doses of each APEC strain (Additional file 1: Fig. S1). The LD of APEC O1, O2 and O78 in this model was 5 × 10^8^, 1 × 10^9^ and 5 × 10^8^ CFU, respectively. After vaccination with various doses of the MOMVs every week for 3 weeks (Fig. 6a, upper panel), the chickens were infected with the LD of each APEC strain 1 week after the last vaccination, respectively. During a 10-day observation phase, all of the PBS-immunized birds died 7 days after challenges of APEC O1, O2 and O78, respectively. However, the survival rate of MOMVs-immunized birds was obviously improved in a dose-dependent manner within a certain dose range. Immunization with 50 μg of MOMVs accounted for 100%, 90% and 100% of the protective efficacy against infection of APEC O1, O2 and O78, respectively (Fig. 6a, below panel). However, the protective efficacy was not improved when the immunization dose of MOMVs was further increased to 100 μg. To investigate whether different doses of MOMVs cause any adverse effect on chickens, we examined the effect of MOMVs immunization on growth performance and inflammation-related cells (Additional file 2: Fig. S2). Immunization with 10 and 50 μg of MOMVs had no significant effects on growth performance and the number of blood inflammation-related cells during the immunization period of 7–28 days. However, immunization with 100 μg of MOMVs significantly reduced growth performance and the number of platelets and increased the number of white blood cells, indicating that an occurrence of inflammation in body. Together, it was reasonable to choose 50 μg as the final vaccination dosage in the current study. Next, we evaluated whether MOMVs immunization had a long-term protection against APEC infections. Five weeks after the final immunization (day 56), chickens in the group immunized with 50 μg of MOMVs were infected with the 2× LD of each APEC strain (Fig. 6b). The survival rate of the MOMVs-immunized group was significantly higher than that of the PBS-immunized group after infection of each APEC strain. Collectively, these results suggested that immunization with 50 μg of MOMVs can provide effective cross-protection against infections induced by these three APEC strains.

### MOMVs-mediated protective immunity agreed with the reduction of bacterial burden and inflammatory cytokine production

**Fig. 7:**
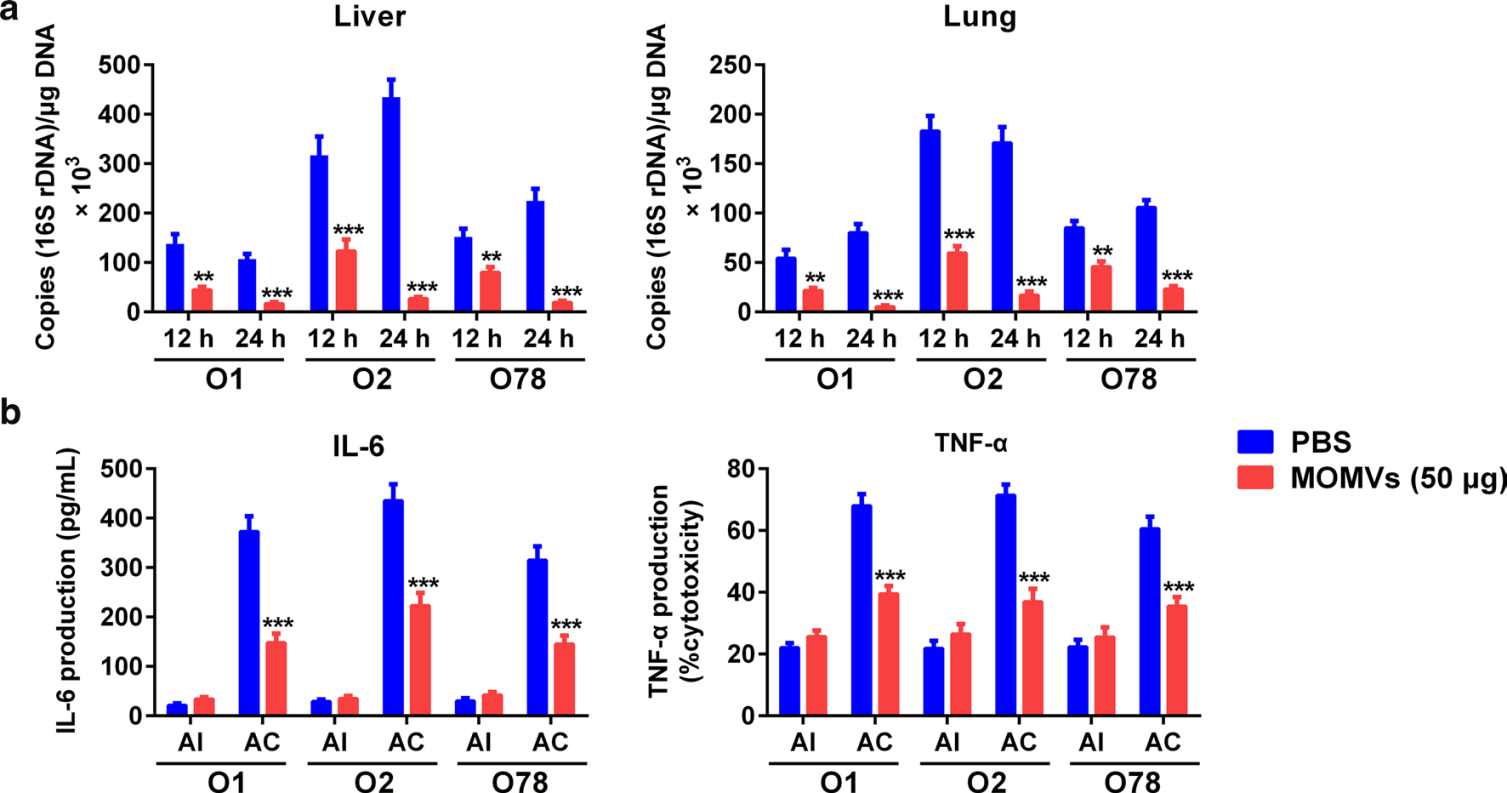
MOMVs-mediated protective immunity agreed with the reduction of bacterial burden and inflammatory cytokine production. **a** Bacterial burdens in liver and lung of chickens immunized with MOMVs (50 μg) and PBS at 12 and 24 h after challenge with the lethal dose of each APEC serogroup (O1, O2 and O78). Bacterial burden was estimated by qRT-PCR using specific primers and probe derived from 16S rDNA sequences of *E. coli* (n = 5). **b** The production of pro-inflammatory cytokines (IL-6 and TNF-α) in serum from MOMVs (50 μg)- and PBS-immunized chickens at day 22 (24 h after the last immunization, AI) and day 29 (24 h after challenge of each APEC serogroup, AC) (n = 5). Data are representative of three independent experiments. **P < 0.01; ***P < 0.001; versus the control (PBS)

In order to study the possible mechanism of MOMVs-mediated protective immunity, we first examined the bacterial burdens in liver and lung tissues from chickens immunized with MOMVs (50 μg) or PBS at indicated time after APEC infection. As shown in Fig. 7a, immunization with MOMVs significantly reduced the counts of APEC strains in both liver and lung tissues, suggesting an effective clearance of these pathogens at 24 h after challenge. These findings can be confirmed by the results of bacterial CFU counting in liver tissue (Additional file 3: Fig. S3). In contrast, a large number of bacteria were found in these two tissues from the PBS-immunized chickens. Then, we determined the levels of major pro-inflammatory cytokines (IL-6 and TNF-α) in the serum from MOMVs- and PBS-immunized chickens sampled at 24 h after the last immunization and 24 h after the bacterial challenge. Similar levels of these pro-inflammatory cytokines were observed between MOMVs- and PBS-immunized groups at 24 h after the last immunization, indicating again that immunization with 50 μg of MOMVs would not induce inflammation (Fig. 7b). Although pathogen challenge significantly improved the production of serum IL-6 and TNF-α in both the MOMVs-immunized group and the control, the levels of these two cytokines were significantly lower in the MOMVs-immunized group. These findings demonstrated that MOMVs mediated protective immunity via reducing bacterial burdens and pro-inflammatory cytokine levels.

### Both vesicular LPS and proteins were key factors in MOMVs-mediated protection

**Fig. 8:**
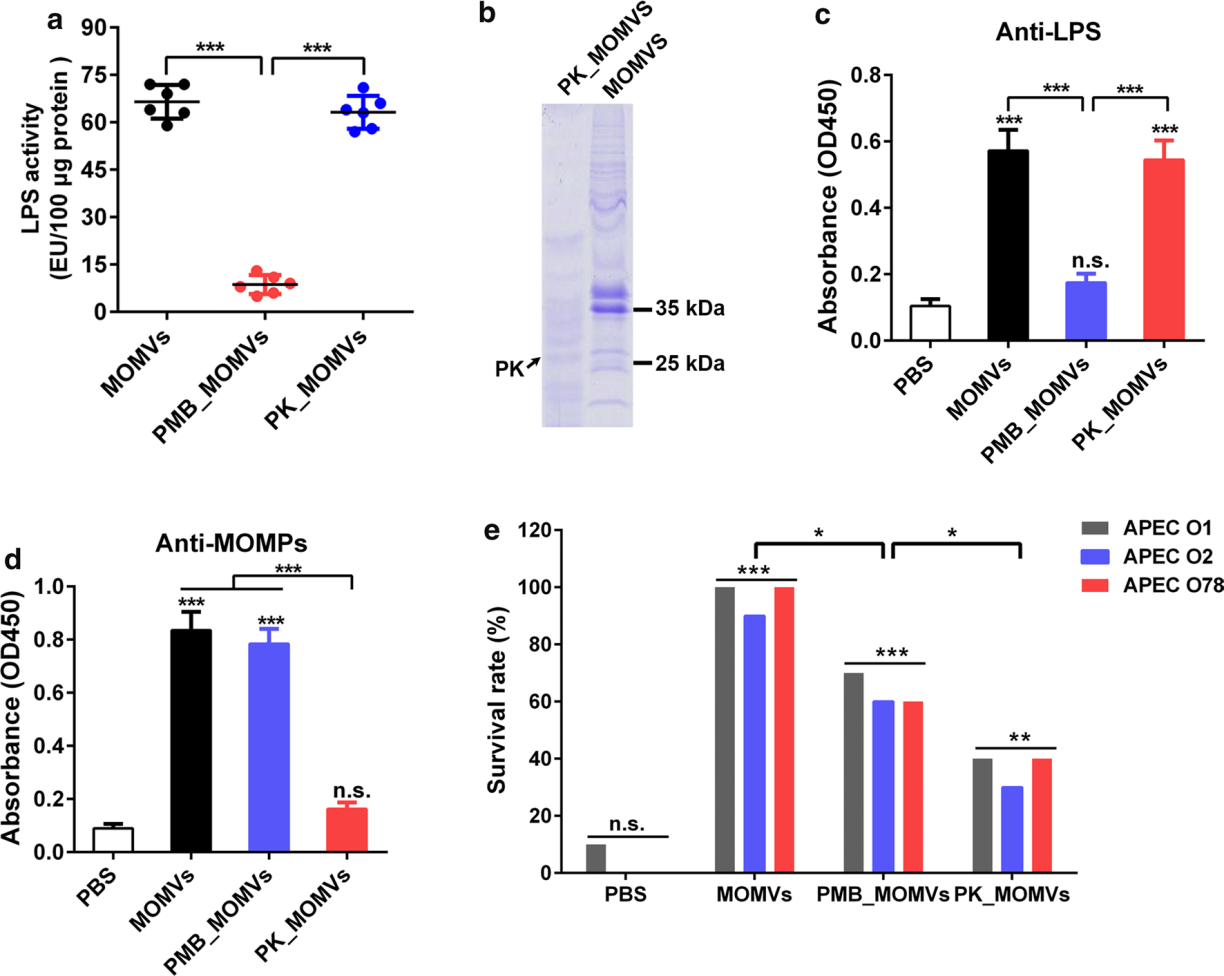
Evaluation of the roles of vesicular proteins and LPS in MOMVs-mediated protection. **a** Determination of LPS contents in MOMVs, polymyxin B-treated MOMVs (PMB_MOMVs) and proteinase K-treated MOMVs (PK_MOMVs) by LAL assay. **b** Coomassie Brilliant Blue-stained SDS-PAGE analysis detected the protein profiles of MOMVs and PK_MOMVs. The IgG titers of anti-OMPs (**c**) and anti-LPS (**d**) in sera from chickens (n = 5) immunized with PBS, MOMVs, PMB_MOMVs and PK_MOMVs. **e** Survival rates of MOMVs- (50 μg), PMB_MOMVs-(50 μg), PK_MOMVs-(50 μg) and PBS-immunized chickens (n = 10) after the lethal infections of these three APEC serogroups (O1, O2 and O78). *P < 0.05; **P < 0.01; ***P < 0.001; n.s., not significant; versus the control (PBS)

Studies have shown that LPS and OMPs enriched in OMVs are known to be potent immunostimulators [28–30]. In the present study, proteomic analysis (Fig. 2) and LAL assay (Fig. 8a) also revealed the abundance of LPS and OMPs in MOMVs. We further investigated the roles of vesicular proteins and LPS in MOMVs-induced protection. MOMVs were treated with polymyxin B (PMB_MOMVs) and proteinase K (PK_MOMVs) to remove vesicular LPS and proteins, respectively. The effectiveness of the treatments was confirmed by LAL assay (Fig. 8a) and SDS-PAGE analysis (Fig. 8b). When chickens were immunized with PMB_MOMVs (50 μg) for 3 weeks, no obvious induction of anti-LPS IgG was observed in the PMB_MOMVs group (Fig. 8c). Similarly, no significant induction of anti-MOMPs IgG in the PK_MOMVs group when chickens were immunized with PK_MOMVs (50 μg) (Fig. 8d). These findings indicated both vesicular LPS and proteins were essential for IgG immune responses. Finally, we evaluated the protection effect of immunization with PMB_MOMVs (50 μg) and PK_MOMVs (50 μg). As shown in Fig. 8e, when chickens were infected with the APEC strains, survival rate of both PMB_MOMVs and PK_MOMVs group was significantly higher than that of the PBS control group, whereas significantly lower than that of the MOMVs group. Moreover, the survival rate of PK_MOMVs group was obviously lower than that of the PMB_MOMVs group. These findings indicated both vesicular proteins and LPS were crucial in MOMVs-mediated protection with vesicular proteins showing a higher protective efficiency.

## Discussion

Despite the discovery of OMVs secreted by Gram-negative bacteria in the 1960s, the composition and function of OMVs have not been studied until the last decade [31, 32]. Therefore, the applications of bacterial OMVs research in many fields are still in its infancy. Recently, OMVs have attracted more and more attentions as new and feasible candidates for the development of next generation vaccines [13]. Many researchers have been trying to develop effective vaccines against APEC infection. To date, as the diversity of APEC serogroups poses challenges for the development of viable vaccines, there are currently no licensed vaccine candidates to prevent APEC infection in poultry farms [9]. To overcome this obstacle, we prepared OMVs from three APEC strains, based on which, a novel multi-serogroup vaccine candidate with multivalent OMVs immunogen was designed, and then, the protective immunity induced by the MOMVs in chickens was investigated. The results demonstrated that the MOMVs could effectively protect chickens against lethal infections induced by multi-serogroup APEC strains. Both innate and adaptive immune responses activated by MOMVs were involved in the cross-protection.

In order to develop an ideal vaccine against APEC infection, several factors have to be taken into account, including the method of vaccination, the use of the adjuvant, the safety and stability and the broad protection [9]. Since mass vaccination is required to immunize a large number of chickens, we focused on vaccination via intramuscular injection, which is the most common route of chicken immunization. The use of adjuvants is an important factor affecting the protection efficacy of vaccines. Vaccine adjuvants play a very important role in the quality and intensity of immune responses [33]. Many Toll-like receptors (TLR) agonists are commonly used as immune adjuvants to enhance antigen-specific immune responses [34]. OMV_APEC_ contains many components found in the outer membrane of their parental bacteria, which are ligands for TLR, thus making OMV_APEC_ itself a good adjuvant [23, 35]. In the present study, the good immunogenicity of MOMVs was demonstrated by their roles in inducing innate and adaptive immune responses. Although OMVs carry some components of virulence factors, numerous studies have shown the safety of low-dose OMVs [15, 17, 22], which is also confirmed in our experiments. The unique structural characteristics with nano-scale vesicles and spherical lipid-bilayers endow OMVs as a biocompatible and stable vaccine carrier [36]. Moreover, an ideal APEC vaccine must be capable of eliciting cross-protection to against various APEC serogroups. OMVs from a single serogroup can induce certain cross-protection because they contain a wide variety of conserved immunogenic components, including a large number of OMPs and pathogen-related molecular patterns [11, 14, 37]. Recent study has showed that immunization with OMVs produced by APEC O78 can protect chickens from APEC O78 infection [27]. However, a single serogroup OMVs may be difficult to induce effective protection against APEC multi-serogroups. To improve the protective efficacy, we developed the MOMVs vaccine with three different OMVs from APEC serogroups (O1, O2 and O78) that frequently cause APEC infection. The broad and long-term effect of the MOMVs also was verified in our animal experiments.

In addition to the improved survival rate, the treatment of MOMVs led to significant pathogen clearance and reduction of pro-inflammatory cytokines, confirming the effective protection of MOMVs. Many studies have been published to explain the mechanism of MOMVs in the treatment of pathogenic *E. coli* infection. Several studies revealed the importance of innate immune cells, such as neutrophils and macrophages [38]. Many studies demonstrated that specific antibody responses played the dominant role [26, 39, 40], while some reports emphasized the importance of the cellular immune response, especially Th1- and Th17-mediated immune responses [22]. In fact, both antigen-nonspecific innate immunity and antigen-specific adaptive immunity cooperated in host defense against pathogen invasion. Macrophages play a connecting role between innate and adaptive immunity by presenting antigen and initiating antigen-specific immune responses via the secreted cytokines [41]. We found that MOMVs were internalized by chicken macrophages and these cells were provoked to secrete Th1- and Th17-polarizing cytokines (IL-12 and IL-6) in vitro. Consistent with our findings, previous studies have revealed that bacterial OMVs can activate innate immune cells, such as dendritic cells and macrophages, to produce immunoregulatory cytokines and co-stimulatory molecules [17, 22]. Besides, MHC-II molecules can deliver exogenous antigens to Th cells, triggering the activation of these cells and thus inducing adaptive immune responses [42]. After immunization with MOMVs, the expression of MHC-IIβ was obviously elevated in the spleen of MOMVs-immunized birds, indicating that MOMVs were recognized by APCs, and antigen presentation was enhanced. Studies have shown that bacterial OMVs can induce specific antibodies and T cell immune responses [15–17, 22]. Our animal experiments also showed that MOMVs vaccination improved the production of specific antibodies and expression of Th1- and Th17-mediated immune genes (IFN-γ and IL-17), indicating that both specific humoral and cellular immune responses were involved in the protective immunity induced by MOMVs. Due to the fact that vesicular proteins account for the highest proportion of the whole vesicle, and OMPs mainly contribute to the humoral immune response, we suspected that humoral immunity might be the major contributor to the MOMVs-mediated protection. The assumption was supported by the results that immunization with protein-deficient MOMVs dramatically reduced the survival rate of chickens infected by APEC strains. However, this does not necessarily mean that MOMVs work independently of cellular immunity, as APEC strains cause systemic infections, suggesting that cellular immunity may also play an important role in combating APEC infection [43]. Further studies are needed to determine the importance of humoral and cellular immune responses in MOMVs-induced protection.

Although the cross-protective effect of MOMVs was confirmed in the present study, it is difficult to clarify which components of the MOMVs are the main factors in immunoprotection [37]. Among the components of the OMVs, protein is the most important composition and mediates many functions of OMVs [11]. Proteomic studies have shown that OMPs are the most abundant molecules of total OMVs proteins, followed by cytoplasmic proteins, while periplasmic proteins and inner membrane proteins are the least. Most of these OMPs work as protective antigen and strongly provoke T cell-dependent humoral immune responses [28, 44]. The immunostimulation and protective effects of these proteins have been reported in various studies [28, 45, 46]. Several major OMPs, including OmpA, OmpC and OmpF, were identified in OMVs derived from APEC strains and other extraintestinal pathogenic *E. coli* [46, 47]. In particular, OmpA and OmpC are ubiquitously found in all *E. coli* strains and have the ability to elicit protective immunity against pathogenic *E. coli* [46, 48]. These conserved OMPs provide a certain level of cross-protection in various pathogenic *E. coli* strains. In addition, some T cell-independent antigens, such as LPS, are also responsible for the protective effects of OMVs [49]. Compared with OMPs, LPS only accounts for a small fraction of OMVs. As a T cell-independent antigen, LPS does not induce a high level of specific antibody response. Considering that vesicular proteins were more protective than LPS, OMPs might be the main composition that provoked cross-protection in MOMVs. However, it does not mean that those non-protein antigens are not essential for the protective effects of MOMVs. The combination of these protective antigens makes MOMVs broadly immunogenic. Further studies are needed to explore the exact components involved in the protective effects of MOMVs.

Compared with other traditional APEC vaccines developed over the years, the MOMVs-based vaccines were naturally obtained from bacteria and has many advantages. First, the MOMV-based vaccines are easy to obtain from bacteria. Second, because of the nanostructures and biocompatibility, MOMV vaccines have the potential for both immunoadjuvants and antigen delivery platforms [14]. Finally, and perhaps most importantly, MOMV-based vaccines may overcome the inefficiency of current APEC vaccines due to the broad immunogenicity of MOMVs derived from the diversity of both immunogens in individual OMV_APEC_ and OMV_APEC_ serogroups [11, 13]. Although our work is encouraging, there are still many limitations. MOMVs vaccines may be more costly because of their time-consuming preparation and relatively low production, which is the limiting factor for the current large-scale application of OMVs vaccines. Besides, the window between effect and toxicity of MOMVs vaccines seems small. Further study is needed to reduce endotoxicity and production cost to make MOMVs vaccines become more convenient. Here we believe that the bionic OMVs solution, such as bacterial spheroplast-derived nanovesicles, may be a better choice [50]. This bacterial spheroplast-derived nanovesicles can be obtained from bacterial spheroplast using a series of extruded procedures, which contains relatively few components of cell-wall toxins and can be adopted in mass production. Therefore, these bionic OMVs vaccines may be both effective and lower side-effects.

## Conclusions

To the best of our knowledge, the current APEC vaccines are more or less problematic in terms of poor safety and low efficacy. Here, we developed a multi-serogroup OMV_APEC_ formulation to obtain broad-spectrum and long-term protection against multi-serogroup APEC infection. MOMVs vaccination provides broad protection via a combined humoral and cellular immunity. Although the detailed mechanisms of MOMVs-mediated cross-protection still need further investigation, our present work provides a new idea for the development of APEC vaccines against multi-serogroup APEC outbreaks. MOMVs vaccines could be used as candidates for next generation APEC vaccines due to their advantages, such as low toxicity and broad protection.

## Methods

### Animal and housing

Experimental procedures and animal use were approved by the Northwest A&F University Animal Care and Use Committee. Arbor Acres broiler chickens were purchased from Dacheng Poultry Industry Company (Xianyang, China) and raised in clean and sterilized rooms under standard conditions until they were 7 days old. Each room was provided with filtered, non-circulated air, and air pressure differences and strict sanitary conditions were maintained.

### Bacterial strains and preparation of MOMVs

Three most common APEC strains that cause chicken colibacillosis, including O1, O2 and O78 serogroups, were obtained from China Veterinary Culture Collection Center. Bacterial OMVs were prepared from these three APEC strains using the protocol as described previously [51, 52]. Briefly, the bacterial strain was grown in LB to the logarithmic phase at 37 °C shaking at 180 rpm. Bacteria-free supernatant was collected by centrifugation (15 min, 12,000*g*, 4 °C) and then filtered through a 0.45-μm bottle top vacuum filter (Corning, NY, USA). The filtered supernatant was concentrated using an Amicon Ultrafiltration system (Merck Millipore, Billerica, Massachusetts, USA) with a 100 kDa-exclusion filter, and subsequently subjected to ultracentrifugation (2 h, 150,000*g*, 4 °C) in a Beckman type 70 Ti rotor (Beckman, CA, USA). The pellet containing OMVs was resuspended in sterile PBS (pH 7.4) and further purified by OptiPrep density gradient centrifugation (16 h, 180,000*g*, 4 °C) with Optiprep (Sigma-Aldrich) concentrations ranging from 10% to 55% (w/v) [26]. After centrifugation, each fraction from the top of the gradient to the bottom was collected to determine the particle number by nanoparticle tracking analysis (NTA). These fractions enriched with OMVs were pooled, diluted in sterile PBS and then centrifuged (2 h, 150,000*g*, 4 °C) to remove OptiPrep. Purified OMVs pellet was resuspended in sterile PBS, sterilized by filtration (0.45 μm; Millipore, Bedford, MA), and finally stored at − 80 °C until future use.

The protein concentration of OMVs was measured by a bicinchoninic acid assay kit (Nanjing Jiancheng Bioengineering Institute, Jiangsu, China). The purified OMVs from these three APEC strains were uniformly mixed in equal proportions to formulate the final MOMVs. Outer membrane proteins (OMPs) were prepared from each APEC strain using the Sarkosyl method as described previously [53]. Multi-serogroup OMPs (MOMPs) were formulated in the same way as MOMVs and used to measure the anti-MOMPs IgG titer in sera. To remove vesicular lipopolysaccharide (LPS) and proteins, native MOMVs were treated with an equal amount of polymyxin B (PMB_MOMVs) and 50 μg/mL (3 U/mL) proteinase K (PK_MOMVs) according to previously reported methods, respectively [54, 55]. Inactivation of proteinase K was performed by raising the temperature (75 °C for 30 min) and adding proteinase K inhibitor (Cocktail Set I, Sigma-Aldrich). Limulus Amebocyte Lysate (LAL) and SDS-PAGE electrophoresis assays were performed to confirm the effectiveness of the treatments. These MOMVs were used for subsequent vaccination of chickens.

### Characterization and proteomic analysis of MOMVs

Purified MOMVs were visualized to detect their morphology and integrity by scanning electron microscopy and transmission electron microscopy using a Field Emission Scanning Electron Microscope (S-4800, Hitachi, Tokyo, Japan) and FEI Tecnai™ G2 Spirit BioTWIN (FEI Company, OR, USA), as described previously [56]. The diameter size distribution of MOMVs was assessed by NTA using a Nanoparticle Analyser (NanoSight, Malvern, Worchestershire, UK) with the operating parameters as follows: 15 for camera level, five 60-s videos for each sample and 6 for detection threshold. To determine the proteome of MOMVs, proteins (10 μg) of MOMVs were separated by 10% SDS-PAGE gel followed by staining with Coomassie Brilliant blue G250 (Sigma-Aldrich). Protein lanes were extracted from the gel, and then digested with trypsin. The obtained peptides were analyzed by the UPLC coupled to tandem mass spectrometry (MS/MS) (LC–MS/MS; Thermo Scientific) [57]. The resulting MS/MS data from three independent experiments were processed separately using Maxquant search engine (v.1.5.2.8). For protein identification, mass spectra were matched with typical *E. coli* K-12 strains in the UniProt database. All searches were filtered using the parameter settings described in a previous study [57]. The identified proteins were analyzed by subcellular localization as well as Gene Ontology (GO) biological processes and molecular functions using CELLO (http://cello.life.nctu.edu.tw/) and InterProScan (http://www.ebi.ac.uk/interpro/), respectively.

### In vitro studies of chicken macrophage

The HD11 cells, a transformed chicken macrophage cell line, were used to investigate whether MOMVs could induce innate immune responses in vitro. We first explored the uptake of MOMVs by HD11 macrophages using a co-culture experiment as described previously [17]. Briefly, dialkylcarbocyanine iodide (DiI, Sigma-Aldrich)-labeled MOMVs were co-cultured with HDl1 cells in complete PRMI-1640 medium (Gibco) containing 10% heat-inactivated FBS (HyClone) and antibiotics (100 U/mL penicillin and 100 μg/mL streptomycin, Sigma-Aldrich) at 37 °C in a 5% CO_2_ atmosphere. After incubation, the cell nucleus was stained with 4, 6-diamidino-2-phenylindole (DAPI, Sigma-Aldrich) and then visualized with High-speed spinning-disk confocal microscope (Andor Revolution XD, Andor Technology, UK). The cells that were not treated with MOMVs were used as the control. We next performed a stimulation assay to evaluate the immune responses of chicken macrophage to MOMVs. HD11 monolayers (1 × 10^6^ cells/mL) were cultured with various doses of MOMVs (0–100 ng/mL) in cell culture medium described above. After 16-h stimulation, the cell culture supernatants were collected for determining the production of cytokines.

### Determination of the lethal doses for APEC strains

Three doses (1 × 10^8^, 5 × 10^8^ and 1 × 10^9^ CFU) of each APEC strain in 100 μL PBS were administrated into chickens by the intratracheal route to determine the lethal dose (LD). The survival rate was recorded every day for 10 days.

### Immunization and challenge

Prior to conducting animal experiments, specific PCR tests were used to ensure that the chickens were not infected with these three APEC strains [58]. To investigate the cross-protective efficacy of MOMVs immunization against APEC infections, 7-day-old chickens were vaccinated three times with 10, 50 and 100 μg of MOMVs in 100 μL PBS at a 1-week interval via the intramuscular route, respectively (Fig. 6a, upper panel). Seven days after the third vaccination (day 28), the birds were infected with the LD of each APEC strain by the intratracheal route. The survival rate was monitored every day for 10 days. To examine the long-term protective effect of MOMVs immunization, chickens were immunized with an optimal dose of MOMVs, and then infected with 2× LD of each APEC strain 5 weeks after the last immunization (day 56). To evaluate the role of vesicular proteins and LPS in MOMVs-mediated protection, we used PMB_MOMVs and PK_MOMVs to immunize chickens, respectively, and observed the survival rate after infection with the LD of each APEC strain.

### Determination of specific antibody titer

One week after each vaccination, sera from chickens were sampled for determining the levels of specific IgG against these three OMVs of the mixed MOMVs using an indirect ELISA method as described previously [22]. Briefly, the 96-well plates were coated with 200 ng of each OMVs overnight at 4 °C and then blocked with 1% bull serum albumin. The sera were diluted by 200-fold in PBS, and used as the primary antibody, which was then added in the blocked wells and incubated at 37 °C for 1 h. The specific IgG was detected after the plates were incubated with secondary HRP-conjugated rabbit anti-chicken IgG (Sigma-Aldrich) followed by the addition of tetramethylbenzidine substrate. The absorbance at 450 nm was detected using a Microplate Reader (Epoch 2, Biotek, Winooski, USA). Each sample was detected in triplicate. The anti-LPS and anti-MOMPs IgG titer in sera were determined by the same method using purified LPS and MOMPs.

### Expression of immune genes

Total RNA was extracted from spleen tissues of MOMVs- and PBS-immunized birds at 1 week after the third immunization using a total RNA kit I (Omega BioTek, Norcross, GA, USA), and then reverse-transcribed into cDNA using the PrimeScript™ RT Reagent Kit with gDNA Eraser (TaKaRa Biotechnology, Dalian, China). Quantitative real-time PCR (qRT-PCR) for immune-related genes (Table 1) was performed in a Real-Time PCR Detection System (CFX96 Touch, Bio-Rad, Hercules, CA, USA). The primers for the target genes and reference gene (β-actin) are listed in Additional file 4: Table S1. Each PCR reaction was conducted in triplicate, as follows: 95 °C for 1 min, 40 cycles of 95 °C for 15 s and 60 °C for 30 s. Relative gene expression was presented as fold-change compared with the control using the 2^−ΔΔCt^ method [59].

### Growth performance and blood parameters

One week after the final immunization, daily feed intake (DFI), average daily weight gain (ADG), feed conversion ratio (FCR) and mortality for the entire period of immunization (days 7–28) were measured as described previously [60]. Blood samples were collected from MOMVs- and PBS-immunized chickens for determination of the number of white blood cells and platelets using an automatic blood cell analyzer (XFA6100; Perlong new technology Co., Ltd., Nanjing, China).

### Measurement of bacterial burden

After APEC infection, bacterial burdens of liver and lung tissues were estimated at indicated times by qRT-PCR as described previously [61]. Briefly, DNA was isolated and purified from liver or lung tissues using a QIAamp DNA Kit (Qiagen, Shanghai, China), and bacterial burden was detected by using specific primers and a probe derived from 16S rDNA sequences of *E. coli*, including the forward primer (5′-CATGCCGCGTGTATGAAGAA-3′), the reverse primer (5′CGGGTAACGTCAATGAG CAAA-3′), and the detecting probe (5′-TATTAACTTTACTCCCTTCCTCCCCGCTGA A-3′). Bacterial burden was presented as the number of 16S rDNA gene copies per unit of total DNA after normalization of total DNA content per unit of tissue for the same sample.

### Measurement of chicken cytokines

The cytokine levels in serum and cell-culture supernatant were determined, including interleukin (IL)-6 and tumor necrosis factor-alpha (TNF-α) in serum collected at day 22 (24 h after the last immunization) and day 29 (24 h after challenge), IL-6, TNF-α and IL-12 in the supernatant of HD11 cells. Since chicken IL-12 has functional homologue and bioactive similarity with human IL-12, we used a human IL-12 ELISA kit (R&D System) to determine chicken IL-12 levels [62]. The production of chicken IL-6 and TNF-α were estimated using IL-6 and TNF-α activity bioassays, respectively [63].

### Statistical analysis

Graph Pad Prism software 5.0 was used for data analysis. Data are shown as the mean ± standard error of the mean (SEM). Student’s *t*-test was used for pairwise comparisons. Significant differences (*P *< 0.05) of means among three or more groups were analyzed using one-way ANOVA with the Newman–Keuls test as the post hoc test. The survival rates after bacteria challenge were compared by the log-rank test.

## Supplementary information

**Additional file 1: Fig. S1.** Determination of the lethal dose of these three APEC serogroups (O1, O2 and O78). Different doses (1 × 10^8^, 5 × 10^8^ and 1 × 10^9^ CFU) of APEC strains were injected by the intratracheal route into chickens (n = 10). Survival rates were recorded every day for 10 days.

**Additional file 2: Fig. S2.** Evaluation of the potential adverse effects from immunization with MOMVs. **a** Effect of immunization with MOMVs on growth performance of each group (n = 20) during the immunization period of 7–28 days, including daily feed intake, average daily weight gain and feed/gain ratio. **b** Effect of immunization with MOMVs on the number of white blood cells and platelets in blood from MOMVs- and PBS-immunized chickens at 7 days after the final immunization (n = 5). *P < 0.05; **P < 0.01; ***P < 0.001; n.s., not significant; versus the control (PBS).

**Additional file 3: Fig. S3.** Bacterial CFU counting in liver tissue from MOMVs- and PBS immunized chickens. The liver samples were homogenized and then prepared with tenfold serial dilutions in sterile PBS and plated on LB agars in triplicate. The colonies were counted after overnight incubation at 37 °C.

**Additional file 4: Table S1.** Primers used for real-time PCR of immune genes in broiler chicken.

## Data Availability

All data generated or analyzed during this study are included in this published article and its additional file.

## Abbreviations

APEC: Avian pathogenic *Escherichia coli*
*E. col*: *Escherichia coli*
OMVs: Outer membrane vesicles
OMV_APEC_: Outer membrane vesicles produced by avian pathogenic *Escherichia coli*
OMV_EC_: Outer membrane vesicles produced by *Escherichia coli*
MOMVs: Multi-serogroup outer membrane vesicles
OMPs: Outer membrane proteins
MOMPs: Multi-serogroup outer membrane proteins
LPS: Lipopolysaccharide
PMB_MOMVs: Polymyxin B-treated MOMVs
PK_MOMVs: Proteinase K-treated MOMVs
NTA: Nanoparticle tracking analysis
LAL: Limulus amebocyte lysate
LD: Lethal dose
DFI: Daily feed intake
ADG: Average daily weight gain
FCR: Feed conversion ratio
