# Supplementary figures and images for: Exploiting bacterial outer membrane vesicles as a cross-protective vaccine candidate against avian pathogenic Escherichia coli (APEC)
Source: Microb Cell Fact. 2020 Jun 3;19:119. doi: 10.1186/s12934-020-01372-7 (PMC7268718; doi:10.1186/s12934-020-01372-7)

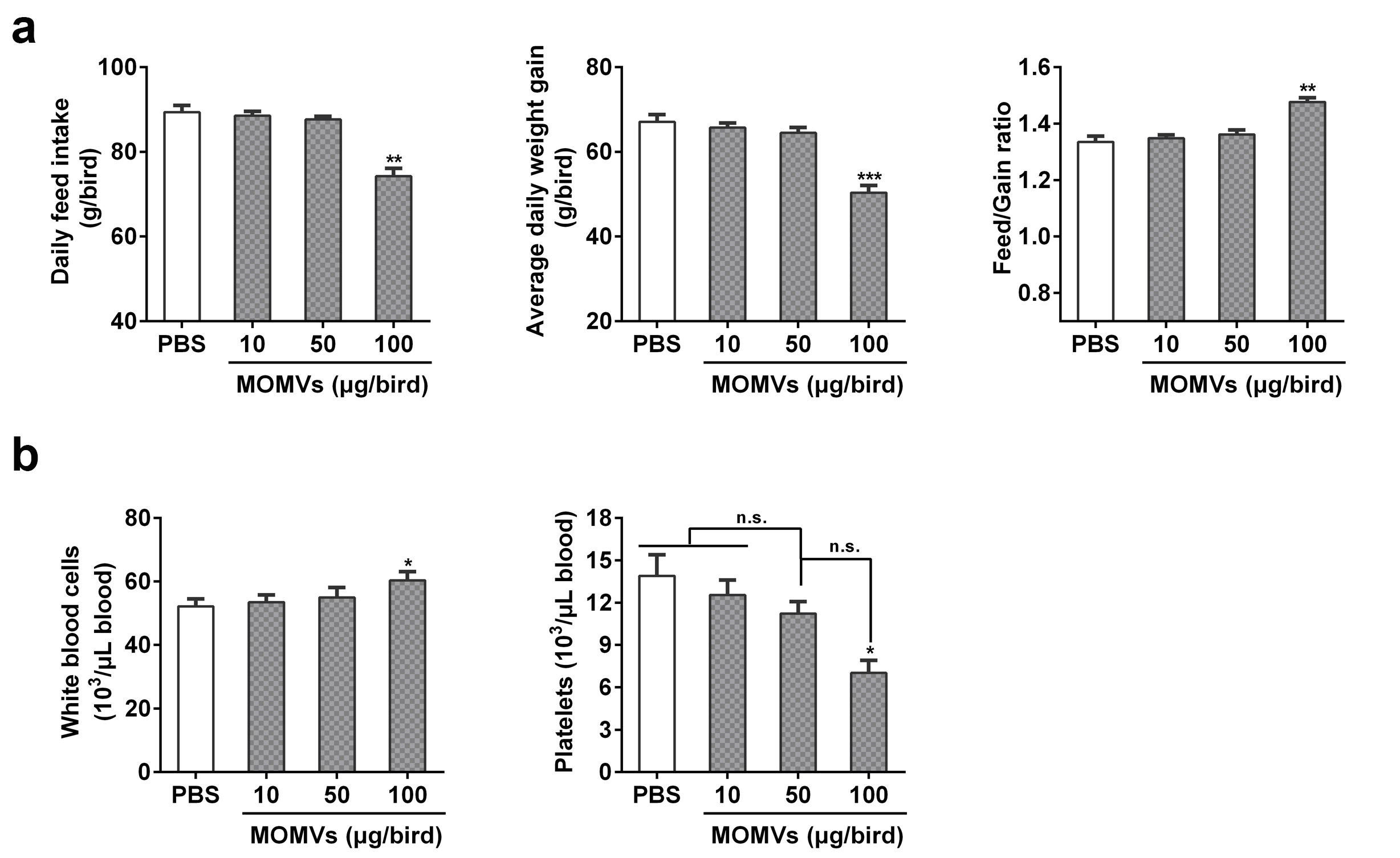

Supplement: Supplementary file 2 — Additional file 2: Fig. S2. Evaluation of the potential adverse effects from immunization with MOMVs. a Effect of immunization with MOMVs on growth performance of each group (n = 20) during the immunization period of 7–28 days, including daily feed intake, average daily weight gain and feed/gain ratio. b Effect of immunization with MOMVs on the number of white blood cells and platelets in blood from MOMVs- and PBS-immunized chickens at 7 days after the final immunization (n = 5). *P < 0.05; **P < 0.01; ***P < 0.001; n.s., not significant; versus the control (PBS). [file 12934_2020_1372_MOESM2_ESM.tif]
