# Supplementary material for: Exploiting bacterial outer membrane vesicles as a cross-protective vaccine candidate against avian pathogenic Escherichia coli (APEC)
Source: Microb Cell Fact. 2020 Jun 3;19:119. doi: 10.1186/s12934-020-01372-7 (PMC7268718; doi:10.1186/s12934-020-01372-7)
Supplement: Supplementary file 4 — Additional file 4: Table S1. Primers used for real-time PCR of immune genes in broiler chicken. [file 12934_2020_1372_MOESM4_ESM.docx]

**Table S1.** Primers used for real-time PCR of immune genes in broiler chicken

| Type^a^ | Gene^b^ | Genebank Number | Primers Sequence (5‘-3’)^c^ | Reference |
| --- | --- | --- | --- | --- |
| Reference | β-actin | L08165 | F: ATTGTCCACCGCAAATGCTTC | Shen et al., 2016 [1] |
|  |  |  | R: AAATAAAGCCATGCCAATCTCGTC |  |
| Th1 | IFN-γ | NM_205149 | F: AGCTGACGGTGGACCTATTATT | Lee et al., 2010 [2] |
|  |  |  | R: GGCTTTGCGCTGGATTC |  |
| Th2 | IL-4 | NM_001007079 | F: TGTGCCCACGCTGTGCTTACA | Waititu et al., 2014 [3] |
|  |  |  | R: CTTGTGGCAGTGCTGGCTCTCC |  |
| Treg | IL-10 | NM_001004414 | F: CGGGAGCTGAGGGTGAA | Lee et al., 2010 [2] |
|  |  |  | R: GTGAAGAAGCGGTGACAGC |  |
| MHC | MHC-IIβ | NM001318995 | F: CCCTCGGCGTTCTTCTTCTAC | Lian et al., 2010 [4] |
|  |  |  | R: CCCACGTCGCTGTCGAA |  |

^a^Th = T helper cell; Treg = regulatory T cell, MHC = major histocompatibility complex.

^b^IL = interleukin; IFN = intrferon.

^c^F = forward primer; R = reverse primer.

1. Shen J, Liu Y, Ren X, Gao K, Li Y, Li S, Yao J, Yang X. Changes in DNA methylation and chromatin structure of pro-inflammatory cytokines stimulated by LPS in broiler peripheral blood mononuclear cells**.** Poultry Sci. 2016;95**:**1636-45.

2. Lee S-H, Lillehoj HS, Jang SI, Hong Y-H, Min W, Lillehoj EP, Yancey RJ, Dominowski P. Embryo vaccination of chickens using a novel adjuvant formulation stimulates protective immunity against Eimeria maxima infection**.** Vaccine. 2010;28**:**7774-8.

3. Waititu SM, Yitbarek A, Matini E, Echeverry H, Kiarie E, Rodriguez-Lecompte JC, Nyachoti CM. Effect of supplementing direct-fed microbials on broiler performance, nutrient digestibilities, and immune responses1**.** Poultry Sci. 2014;93**:**625-35.

4. Lian L, Qu LJ, Zheng JX, Liu CJ, Zhang YP, Chen YM, Xu GY, Yang N. Expression profiles of genes within a subregion of chicken major histocompatibility complex B in spleen after Marek's disease virus infection**.** Poultry Sci. 2010;89**:**2123-9.
